# Supplementary material for: The Down-Shifting Luminescence of Rare-Earth Nanoparticles for Multimodal Imaging and Photothermal Therapy of Breast Cancer
Source: Biology (Basel). 2024 Feb 28;13(3):156. doi: 10.3390/biology13030156 (PMC10967896; doi:10.3390/biology13030156)
Supplement: Supplementary file 1 [file biology-13-00156-s001.zip › biology-2856727-supplementary.pdf]

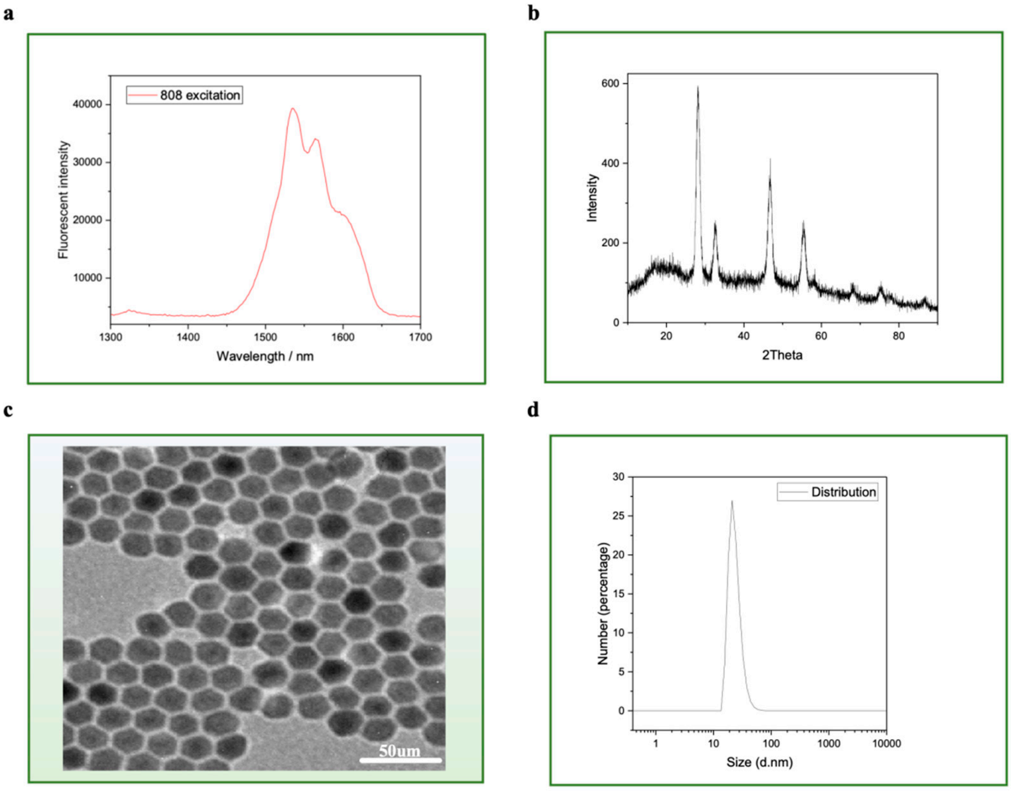

Supplementary Figure S1. Characterization of α-Er NPs. a,The NIR-II fluorescence spectrum of α-Er NPs. b,The size and position of the main diffraction peaks in the XRD pattern of α-Er NPs. c,Transmission electron microscope (TEM) images of α-Er NPs. d,Size distribution of α-Er NPs determined by dynamic light scattering.
